# Fees, and How to Collect Them

**Published:** 1898-10

**Authors:** A. B. Freeman

**Affiliations:** Chicago.


ARTICLE IV.

### FEES, AND HOW TO COLLECT THEM.

DR. A. B. FREEMAN, CHICAGO.

The stock in trade or capital of a professional man is not alone the office equipment and material which he may use in conducting his business, but rather the long years of patient toil, study and expense necessary to fit him for practice, the technique, education of brain and muscle which make the services of the successful practitioner valuable to his clients.

Not the least valuable item on the inventory of the dentist's stock is his precious moments—sixty of which make an hour—for every one of which he should receive compensation when reserved for patrons and the appointment is broken without reasonable notice—and reasonable notice is not one minute or one hour.

The fees charged should, therefore, be reasonably commensurate with the foregoing facts and the substantial character of the service rendered.

As a class, doctors are acknowledged to be poor business managers, and not far sighted beyond the ken of their daily routine. At the operating chair and the sick bed their judgment is keen, but beyond giving their best thinking powers to their patrons and matters of a professional nature their keen perceptive faculties seem to fail them, and their surplus earnings, if they are so fortunate as to have them, are often invested in rosy-tinted schemes and trusted to the management of others, where conservative business men would hesitate to place them. The result is losses which can never be regained.

If there be an individual in any vocation of life, either commercial, agricultural or professional, who deserves just remuneration for his services or a comfortable competency in his old age, I assert it is the dental practitioner. For, in addition to all the intimate relations and attentive services of the medical practitioner, do we not advance quite a large proportion of cash to each patron to render our services?

It is a notorious fact that in all great metropolitan centers like Chicago quite a large percentage of the population, comparatively speaking, is made up of impecunious persons, either of the improvident or dishonest class, the one contracting debts without thought of how the bills are to be met, and the other class with only the one intention of getting something for nothing.

None of us have objections to the gratuitous treatment of the worthy poor at the free dispensaries or the dental clinics, and most of us willingly and gratuitously contribute to the comfort of many of our unfortunate patrons and friends each year, but we should draw the line at impositions.

Heretofore each victimized practitioner has considered imposition to be one of the inevitable results, and while he has probably never allowed himself to be imposed on a second time by the same individual he has never lifted a finger in protection of his professional brothers, while the same dead beat has continued to practice his evil art whenever necessity demanded.

It is not an alluring prospect which confronts us as we scan our accounts from year to year and note, with dissatisfaction, that we have been victimized by from six to ten new imposters within a twelve month.

The average annual tribute which I have personally paid this fraternity for the ten years last past is trivial indeed, and yet it amounts to $1,200 for the term.

Admitting the proposition, as we must, that as a profession we have no adequate protection against the dead-beat evil, the question naturally arises, why not ?

The chief reason would seem to be lack of earnest co-operation organization, in the management of which the profession at large has the utmost confidence.

The benefits to be derived from co-operation, or the association of any number of persons who act conjointly for their common good, are well understood, and no better illustration can be cited than the Dental Protective Association, under the able leadership of Dr. Crouse.

The large commercial interests, both wholesale and retail, are protected by inflexible credit system and reliable information furnished by well-known commercial agencies.

During the past few weeks the real estate brokers and rent collectors of this city and St. Louis have been actively agitating the formation of a rating department, to be conducted by the real estate boards, for the sole purpose of protection against dead-beat tenants.

The retailers of contracting and building supplies have an ideal co-operative organization in this city, and furnishes protection to a large membership, including all departments of supply in this trade.

The physicians of New York City are organized.

The physicians of Chicago chartered and organized the Physicians' Business Bureau some three years since, and have an admirable scheme for co-operative protection, which has practically been inoperative, chiefly because by far the larger portion of the profession has been indifferent—only one hundred and fifty joining the bureau out of a profession numbering three thousand.

The medical and dental profession in Philadelphia are furnished adequate protection by a private agency with whom they co-operate.

These facts illustrate that in other avenues of industry, as well as in our own, to some extent, there is organization for protection.

The two organizations, the methods of which the writer has familiarized himself, seeming to embody the most desirable features, are the Philadelphia Physicians' and Dentists' Bureau of Information, and the one furnishing the protection for the retailers to the building trades in this city.

Their modus operandi is the same, in the main, and is as follows:

There is a central organization with a nominal membership fee, the members signing an agreement not to furnish information to any one, and to consider all reports as confidential.

The members report names and addresses of all irresponsible parties who have failed to settle their accounts after proper diligence on the part of the creditor.

The central organization furnishes an annually revised list of names, without comment, and advises each subscriber or member that the name of no responsible person is to be found in this list. Monthly, quarterly or semiannual report of names is required, and additional lists mailed as deemed advisable.

When a delinquent settles an old account it is reported that he has redeemed himself, and the name stricken from the list. These two associations differ in but one point. The latter one, on having a dead-beat reported by a member, sends immediate notice to all other members that a brother member "recommends" this party to the association.

The members fully understand what a "recommendation" means, and govern themselves accordingly.

To conclude, it would seem to be worse than useless that there be a multiplication of organizations in the medical and dental professions, as then the best results are partially, if not wholly, lost.

If there could be a combination of all medical and dental interests, and this combination, as a whole, cooperate, and furnish the names of dead-beats, as well as those of very doubtful credit, what could not be accomplished in the way of co-operation and self-protection? —*Review.*

